# Unusual ocular manifestations of ethomoidal mucocele: a case report

**DOI:** 10.5935/0004-2749.2021-0313

**Published:** 2022-09-06

**Authors:** Selim Cevher, Serdar Ali Elkıran

**Affiliations:** 1 Hitit University Faculty of Medicine, Department of Ophthalmology, Corum, Turkey; 2 Hitit University Erol Olçok Training and Research Hospital, Department of Otolaryngology, Corum, Turkey

**Keywords:** Maculopathy, Tomography, optical coherence, Choroid diseases, Mucocele, Maculopatia, Tomografia de coerência óptica, Doenças da coroide, Mucocele

## Abstract

A 42-year-old female patient had vision loss and chronic epiphora in her left eye. Her best-corrected visual acuity was 10/10 in the right eye and 0.3/10 in the left eye. The anterior segment examination results were normal. In fundus examination, choroidal folds were detected. Optical coherence tomography showed elevation on the macula and choroidal folds. Ultrasonography revealed a T-sign. Magnetic resonance imaging revealed an ethmoidal mucocele that compresses the orbital tissues. Surgical treatment was performed in the otorhinolaryngology department. Postoperatively, choroidal folds recovered, and the best-corrected visual acuity improved, but subretinal fluid accumulated. During the follow-up period without any treatment, subretinal fluid totally disappeared.

## Abstract (pt)

Paciente do sexo feminino, 42 anos, com perda visual e epífora crônica no olho esquerdo. Sua acuidade visual melhor corrigida foi de 10/10 no olho direito e 0,3/10 no olho esquerdo. O exame do segmento anterior foi normal. No exame de fundo de olho, foram detectadas pregas coroidais. A tomografia de coerência óptica revelou elevação na mácula e pregas coroidais. A ultrassonografia revelou sinal T. A imagem de ressonância magnética mostrou mucocele etmoidal que comprime os tecidos orbitários. O Departamento de Otorrinolaringologia realizou o tratamento cirúrgico. No pós-operatório, as dobras coroidais se recuperaram, a acuidade visual melhor corrigida foi melhorada, mas ocorreu líquido sub-retiniano. Durante o período de acompanhamento sem qualquer tratamento, o líquido sub-retiniano recuperou-se totalmente.

## INTRODUCTION

Mucoceles are benign lesions that occurred after complete ostial obstruction. Obstructions are caused by trauma, tumor, chronic sinusitis, and mucosal edema^(1,2)^. Mucous secretions accumulate, and cyst formation occurs. Mucoceles can expand slowly, and they can fill the paranasal sinuses completely. They have a potential to invade or compress the orbit^(3)^.

Clinical manifestations vary widely and depend on the location of the mucocele. Ethmoidal mucoceles usually affect the orbit and globe. Their mass effects may cause proptosis, globe displacement, palpable mass, diplopia due to extraocular muscle restriction, epiphora due to the invasion or compression of nasolacrimal passage, eyelid swelling, increasing intraocular pressure, visual loss, and choroidal folds.

Herein, we report a case of ethmoidal mucocele with unusual ocular findings.

## CASE REPORT

The patient was informed about the study and was invited to participate. A signed informed consent form was obtained. The patient was also informed that participation was totally voluntary and that nonparticipation would have no negative effects on her treatment and relationship with her physicians. This study conformed to the tenets of the Declaration of Helsinki.

**Figure 1:**
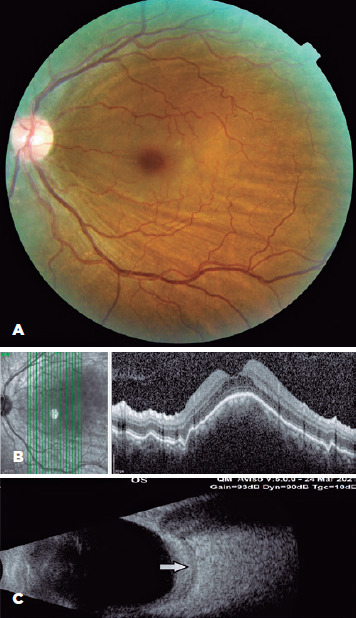
Preoperative findings. (A) Choroidal folds on the fundus. (B) Optical coherence tomography shows macular elevation and choroidal folds. (C) Orbital ultrasonography image of the T-sign (indicated by arrow).

A 42-year-old female patient presented to our clinic with visual loss, pain, and epiphora in her left eye for 3 months. Her best-corrected visual acuity (BVCA) values were 0.0 LogMAR in the right eye and 0.5 LogMAR in the left eye. Anterior segment structures were normal bilaterally. Results of Goldmann applanation tonometry were 18 mmHg and 20 mmHg in her right and left eyes, respectively. During fundus examination, choroidal folds were detected in her left eye (Figure 1A), and her macula was elevated. Fundus examination results of the right eye were normal. She had normal extraocular movement in both eyes. Nasolacrimal lavage of the left eye was blocked. The palpation of the orbital rim might indicate the presence of a mass. She had neither systemic disorders nor history of ocular surgery and retinal disorder.

**Figure 2:**
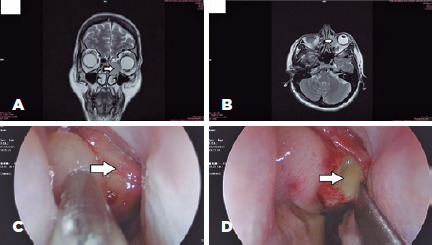
Magnetic resonance imaging and endoscopic findings. Magnetic resonance imaging (A) (coronal scan) shows ethmoidal mucocele (indicated by arrow) (B) (axial scan) shows ethmoidal mucocele (indicated by arrow). (C) Endoscopic view of the mucocele (indicated by arrow). (D) Endoscopic view of the thick yellowish mucin discharge (mucocele; indicated by arrow).

Optical coherence tomography (OCT) revealed macular elevation (Figure 1B). Orbital ultrasonography (USG) detected the T-sign (Figure 1C). An ethmoidal mucocele that compresses the orbital tissues was detected in the magnetic resonance imaging of the orbit (Figures 2A and B). We decided to refer the patient to the department of otorhinolaryngology. Rigid nasoendoscopy revealed left ethmoidal cystic lesion (Figure 2C).

Examinations results indicated an ethmoidal mucocele with orbital compression, and transnasal endoscopic marsupialisation and drainage were performed (Figure 2D).

**Figure 3:**
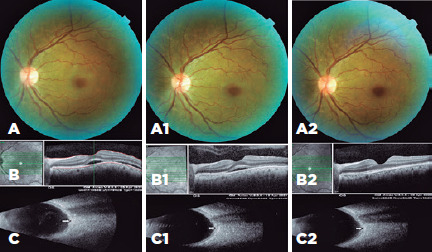
Postoperative findings. (A) Normal fundus image on the first postoperative day. (B) Optical coherence tomography shows subretinal fuid on the first postoperative day. (C) Orbital ultrasonography shows the disappearance of the T-sign (indicated by arrow) on the first postoperative day. (A1) Normal fundus image at the first postoperative week. (B1) Optical coherence tomography shows minimal subretinal fuid at the first postoperative week. (C1) Normal orbital ultrasonography image, showing the disappearance of the T-sign (indicated by arrow) at the first postoperative week. (A2) Normal fundus image at the third postoperative week. (B2) Optical coherence tomography shows totally normal macula at the first postoperative week. (C2) Normal orbital ultrasonography image showing the disappearance of the T-sign (indicated by arrow) at the first postoperative week.

On the first postoperative day, the BCVA was 0.2 LogMAR, choroidal folds recovered (Figure 3A), macular elevation recovered, but subretinal fluid was detected during OCT (Figure 3B), and the T-sign disappeared (Figure 3C).

On the first postoperative week, the BCVA was 0.1 LogMAR, there were no choroidal folds (Figure 3A1), subretinal fluid accumulation decreased but still present (Figure 3B1), and USG findings were normal (Figure 3C1).

On the third postoperative week, the BCVA was 0.0 LogMAR, fundus photography was normal (Figure 3A2), subretinal fluid and choroidal folds totally disappeared (Figure 3B2), and USG findings were normal (Figure 3C2).

## DISCUSSION

Although mucoceles fall within the purview of the otolaryngologist, sometimes they can cause an ocular disturbance, affecting the orbits, and the patients can apply to the ophthalmologist first, as our patient did.w

Ethmoidal mucoceles are the second common mucoceles after frontal mucoceles^(4)^. Mucoceles have expansion capability. They can compress orbital structures and cause orbital complications. According to Tseng et al., the most common ocular manifestations included proptosis, periorbital pain, impaired ocular mobility (mucoceles in the anterior paranasal sinuses), blurred vision, and impaired ocular mobility (mucoceles in the posterior paranasal sinuses)^(5)^. Loo et al. reported orbital manifestations such as proptosis, limited extraocular movements, optic nerve compression, eyelid swelling/erythema, and presence of choroidal folds^(6)^.

The blurred vision is usually associated with optic neuropathy^(7)^. In our patient, the optic nerve was normal, and blurred vision was caused by maculopathy. This case illustrates that mucocele can cause macular elevation, and OCT can help detect this situation. Macular elevation was alleviated after the surgical removal of the mucocele. On the contrary, subretinal fluid accumulation occurred after the first day of the treatment, and it disappeared during the follow-up. We think that the subretinal fluid was associated with hemodynamic and physiological changes. After surgery, the decrease in mucocele pressure to the orbits and the change in the choroidal congestion may cause accumulation of the subretinal fluid. We think that normal physiological functions of the retinal pigment epithelium (RPE) and choriocapillaris recovered with time, and drainage of the subretinal fluid was completed. In the literature, we did not find similar cases caused by ethmoidal mucocele.

Choroidal folds are another sign of mucocele. A wrinkled appearance is caused by the ondulations of structures, such as the choroid, Bruch’s membrane (BM), RPE, and neurosensory retina. However, the pathogenesis of choroidal folds is not fully elucidated. It is usually thought that choroidal folds occur secondary to choroidal congestion^(8)^. Congestion of the choroidal tissue can affect the physiology and changes the shape of the BM, RPE, and neurosensory retina. When the folds involve the macula and/or affect the physiology of the macula, visual acuity may decrease. After surgery, the choroidal folds of our patient disappeared.

In this patient, USG also revealed the T-sign that usually occurs in posterior scleritis, and this sign also improved with a short-term follow-up period. This situation may be associated with the congestion of the posterior pole.

Another clinical finding of our case was epiphora. Chronic epiphora could be caused by the compression of the ethmoidal mucocele to the nasolacrimal duct system.

This patient also presented atypical clinical features. We believe that the clinical and imaging findings of this case are great additions to the literature. Moreover, this case demonstrates that mucoceles may affect both the choroid and retina preoperatively and postoperatively.
